# Surgical management of AAST grades III-V hepatic trauma by Damage control surgery with perihepatic packing and Definitive hepatic repair–single centre experience

**DOI:** 10.1186/s13017-015-0031-8

**Published:** 2015-08-01

**Authors:** Krstina Doklestić, Branislav Stefanović, Pavle Gregorić, Nenad Ivančević, Zlatibor Lončar, Bojan Jovanović, Vesna Bumbaširević, Vasilije Jeremić, Sanja Tomanović Vujadinović, Branislava Stefanović, Nataša Milić, Aleksandar Karamarković

**Affiliations:** Faculty of Medicine, University of Belgrade and Clinical Center of Serbia, Clinic for Emergency Surgery, University of Belgrade, Serbia, Pasteur Str.2, Belgrade, 11000 Serbia; Faculty of Medicine, University of Belgrade and Clinical Center of Serbia, Department for Anesthesiology, University of Belgrade, Serbia, Belgrade, Serbia; Faculty of Medicine, University of Belgrade and Clinical Center of Serbia, Clinic for Physical and Rehabilitation Medicine, Clinical Center of Serbia, Belgrade, Serbia; Faculty of Medicine and Institute for Medical Statistics and Informatics, University of Belgrade, Belgrade, Serbia

**Keywords:** Liver trauma, Damage control surgery (DCS), Hemorrhage, Exsanguinating trauma patients, Mortality

## Abstract

**Background:**

Severe liver injury in trauma patients still accounts for significant morbidity and mortality. Operative techniques in liver trauma are some of the most challenging. They include the broad and complex area, from damage control to liver resection.

**Material and method:**

This is a retrospective study of 121 trauma patients with hepatic trauma American Association for Surgery of Trauma (AAST) grade III–V who have undergone surgery. Indications for surgery include refractory hypotension not responding to resuscitation due to uncontrolled hemorrhage from liver trauma; massive hemoperitonem on Focused assessment by ultrasound for trauma (FAST) and/or Diagnostic peritoneal lavage (DPL) as well as Multislice Computed Tomography (MSCT) findings of the severe liver injury and major vascular injuries with active bleeding.

**Results:**

Non-survivors have significantly higher AAST grade of liver injury and higher Injury Severity Score (ISS) (*p* = 0.000; *p* = 0.0001). Non-survivors have significant hypotension on arrival and lower Glasgow Coma Scale (GCS) on admission (*p* = 0.000; *p* = 0.0001). Definitive hepatic repair was performed in 62(51.2 %) patient. Damage Control, liver packing and planned re-laparotomy after 48 h were used in 59(48.8 %). There was no statistically significant difference in terms of the surgical approach. There was significant difference in the amount of red blood cells (RBC) transfusion in the first 24 h between survivors and non-survivors (*p* = 0.001). Overall mortality rate was 33.1 %. Regarding complications non-survivors had significantly prolonged bleeding and higher rate of Acute respiratory distress syndrome (ARDS) (*p* = 0.0001; *p* = 0.0001), while survivors had significantly higher rate of pleural effusion (*p* = 0.0001).

**Conclusion:**

All efforts in the treatment of severe liver injuries should be directed to the rapid and effective control of bleeding, because uncontrollable hemorrhage is the cause of early death and it requires massive blood transfusion, all of which contributes to the late fatal complication.

## Introduction

Despite the great advances in surgical treatment and resuscitation of trauma patients with liver injuries in the last decades, severe liver trauma still accounts for significant morbidity and mortality [1–4]. Major liver injury is the leading cause of death in patients with abdominal trauma, and their treatment continues to challenge surgeons [5, 6]. The main cause of early liver injury-related death is uncontrolled bleeding, and it is associated with a mortality rate of 50–54 % in the first 24 h after admission, with 80 % of operative deaths [1, 2, 5].

Early diagnosis of the extent of liver trauma with adequate treatment adapted to the severity of the injury and the physiological condition of the patient, may result in significant reduction of morbidity and mortality [5]. Hemodynamic stability is key for diagnostic and therapeutic approach to the severe liver injuries. The diagnosis of hepatic trauma starts simultaneously with reanimation, immediately after admission, which implies targeted clinical examination, laboratory blood tests, abdominal ultrasound (Focused assessment with sonography for trauma, FAST) followed by Multislice Computed Tomography (MSCT) [6–10]. Elevation of the serum aspartate aminotransferase (AST) and alanine aminotransferase (ALT) is the laboratory indicator of liver injury [9].

Menagment of a severe trauma patient involves systematic sequence of actions [8–15]. Haemodynamically unstable patients with major liver injuries require rapid manoeuvers to control bleeding [10]. Accordingly, exsanguinating patients require substantial blood transfusions [11]. Uncontrolled bleeding leads to new adverse events that announce catastrophe–coagulopathy, as a result of depletion and dilution of coagulation factors, acidosis, and hypothermia [10, 11]. The decision for an emergency laparotomy is usually based upon the presence of the “lethal triad” with coagulopathy, acidosis and hypothermia [5, 11]. Surgical treatment in bleeding liver trauma is required in cases of progressive hemodynamic instability due to hemorrhage shock [11]. Operative techniques in liver trauma are some of the most challenging. They include the broad and complex area from Damage control surgery (DCS) to the liver resection [2, 5]. Surgical control of bleeding is the main goal in damage control strategy as well as the prevention of biliary complications which are specific for liver injuries. In unstable patients with severe physiological derangement surgical procedures such as direct vessel repair of juxtahepatic venous injuries and early perihepatic packing with the correction of hypothermia, coagulopathy, and acidosis may lead to improved outcome [5, 10, 11].

The purpose of this study was to determine the predictors of morbidity and mortality in trauma patients that underwent surgery for severe hepatic injury, as well as to identify a better approach for exsanguinating patients.

## Materials and methods

This is the retrospective study of 121 trauma patients with severe hepatic trauma, who have been admitted and operated at Clinic of Emergency Surgery, Clinical Center of Serbia, Belgrade, from November 2008 to January 2015. This study has been performed with the approval of the Ethics Committee of the Clinical Centre of Sebia with a reference number 1533/21.

Severe liver injuries were graded according to the American Association for the surgery of Trauma (AAST) - Organ Injury Scale (OIS) as liver trauma grades III, IV and V [1, 4]. Hemodynamically stable patients who had AAST grade I-II liver injury, treated by Non Operative Management (NOM) were not included in study.

Upon arrival at the emergency room of the trauma patient with uncontrolled hemorrhage, the main goal was identification of the sources of bleeding, followed by prompt bleeding control and resuscitation in order to restore tissue perfusion and to achieve haemodynamic stability. After complete, targeted and very fast clinical examination, Focused assessment with sonography for trauma (FAST) is the first tool used as to see the presence of free fluid (means hemoperitoneum) and associated solid organ injury. In hemodynamically unstable Diagnostic peritoneal lavage (DPL) was performed following a negative FAST scan in the setting of blunt abdominal trauma for rapid diagnosis of abdominal injury requiring emergency laparotomy. Initial MSCT of thorax and abdomen in order to determine the severity of the liver trauma and the presence of associated injuries, was done in haemodynamic stable patients. Shock was defined as a systolic blood pressure of <90 mmHg. Glasgow Coma Scale (GCS) used for evaluation the severity of associated CNS and head injury, by measuring three parameters (motor response, verbal response and eye opening response) range was from 0 (brain death) to the maximum score of 15 for normal cerebral function. The Injury Severity Score (ISS) as an anatomical scoring system providing an overall score for trauma patients with multiple injuries. Each injury is assigned an Abbreviated Injury Scale (AIS) score and is allocated to one of the six body regions (Head, Face, Chest, Abdomen, Extremities (including Pelvis), External). The ISS score takes values from 0 to 75 (lethal injury).

Indications for emergency laparotomy within 30 min uppon trauma patients arrival included: uncontrolled bleeding from liver trauma with positive FAST and/or DPL (hemoperitoneum: blood at initial aspiration or a red blood cell count in the lavage fluid was >100.000/mm^3^); MSCT findings of the massive hemoperitoneum and severe liver trauma with major hepatic vein/VCI laceration, complex perihilar injuries with active bleeding presented as extravasations of intravenous contrast; and hemorrhagic shock with refractory hypotension not responding to initial resuscitation. Laparotomy was performed through midline incision searching for intraperitoneal bleeding, liver trauma, associated abdominal injury and intestinal perforations that call for emergency repair. Blood from the peritoneal cavity was sucked out, folowed by emergency care of intraperitoneal haemorrhage and control of the sources of contamination. Inflow vascular control was employed as Pringle maneuver under vascular clamp before proceeding of liver parenchymal and vascular repair. Direct liver repair techniques have been used as extensive suture (hepatorrhaphy), hepatotomy with selective vascular ligation, selective right hepatic artery (RHA) ligation, resectional de‘bridement and liver resection. Major resection was used only to control extensive laceration of liver and extensive devitalized liver tissue.

In exsanguinating patients with severe physiological derangement (lethal triade) due to exsanguinating liver injuries we used strategy of Damage Control Surgery (DCS). Exsanguination presented the extreme blood loss caused by traumatic complex liver injuries and major blood vessels, with an initial blood loss of >40 % of the entire blood volume. Indications for DCS were:

- Metabolic acidosis (lactic acid level >5 mmol/L, pH <7.2, base deficit >14)
- Hypothermia (core hypothermia <34 °C)
- Coagulopathy (PT and PTT >2 times normal)

Initial emergency laparotomy was the first step of the damage control: fast and limited surgical intervention in order to control life-threatening hemorrhage and control of contamination. We performed perihepatic packing (packing of the liver) with approximately 4–6 abdominal swabs to provide liver compression and bleeding control. Abdominal swabs were never placed directly into the liver laceration and bleeding blood vessels were suture/ ligated prior to liver packing. After liver packing, the abdomen was closed temporarily. From operating theater patient was transferred to the Intensive Care Unit (ICU) for ICU resuscitation and correction of acidosis, coagulopathy, hypothermia, including antibiotic with broad-spectrum aerobic and anaerobic coverage in all patients (DCS II step). Indications for red blood cell (RBC) transfusion included acute blood loss greater than 1500 mL, or 30 percent of blood volume, or hemoglobin level <9 g/dl. Massive transfusion was defined as transfusion of ten or more RBC products within 24 h. The third step in DCS was a planed re-laparotomy and definitive reconstruction. Removal of perihepatic packing and definitive surgical procedure was performed after 48 h when the patient’s temperature has been normalized, shock has been corrected, and the International Normalized Ratio (INR) was less than 1.5.

Data were collected in terms of age, sex, blood pressure on arrival, mechanism of trauma, AAST grades of liver injury, ISS due to severe associated injuries, management and outcome. Types of surgical procedures, RBC transfusion (ml) within first 24 h, Intensive Care Unit (ICU) stay and hospital length of stay were recorded. Liver-related complications were considered to include prolonged massive bleeding (more than 100 ml/h on abdominal drain) despite the surgical control of bleeding, liver failure, bile leak, bile fistula and liver abscess. Biliary leak was defined as any drainage through the abdominal catheter with bilirubin content 2× higher than the plasma levels. The Acute Respiratory Distress Syndrome (ARDS) was defined on three mutually exclusive categories based on degree of hypoxemia: mild (200 mm Hg < PaO_2_/FIO_2_ ≤ 300 mm Hg), moderate (100 mm Hg < PaO_2_/FIO_2_ ≤ 200 mm Hg), and severe (PaO_2_/FIO_2_ ≤ 100 mm Hg) and four ancillary variables for severe ARDS: radiographic severity with bilateral infiltrates, respiratory system compliance (≤40 mL/cm H_2_O), positive end-expiratory pressure (≥10 cm H_2_O), and corrected expired volume per minute (≥10 L/min). Mortality was defined as death within 30 days of hospitalization. Early trauma-related mortality was defined when death occurred within the first 48 h.

### Statistical analysis

Data were analyzed using methods of descriptive and analytical statistics. The methods of descriptive statistics were: measures of central tendency (mean and median), measures of variability (standard deviation and interquartile range) and the relative numbers. The methods of analytical statistics were: identification methods of empirical distributions, methods to assess the significance of differences and Student’s *t* test and rank sum test for numerical variables depending on the normality of distribution and Chi-square and Fisher’s test for categorical variables. Univariate and multivariate logistic regression analysis were used to determine the prognostic factors of mortality with 95 % confidence intervals (CI). For survival analysis was used Kaplan Meier’s survival analysis and Cox’s Proporción hazardous model. A *p* < 0.05 was considered statistically significant.

## Results

**Fig. 1:**
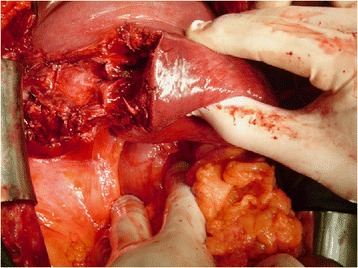
Intraoperative finding in penetrating liver injury. A 36 year old male suffered a penetrating abdominal injury (stab wound to the right upper abdomen) with AAST grade IV liver injury. Non-anatomic liver resection was performed

The general characteristics of all 121 patients with severe liver trauma who were included in our study with comparison between the survivors and non-survivors summarizes in Table 1. In this study 81(66.9 %) patients survived, while 40(33.1 %) of them died. In this study there were 90(74.4 %) males and 31(25.6 %) females (Table 1). Blunt hepatic injury was the leading mechanism of trauma, seen in 98(80.9 %) patients (Table 1). Road traffic accident was the leading cause of blunt trauma recorded in 80 (66.1 %) patients, and among them were 37(30.6 %) drivers, 36 (29.7 %) pedestrians and 7(5.8 %) passengers (data not shown). The remaining 10 (8.2 %) patients with blunt liver trauma were injured by falling from a roof and eight were hiting by assailant (6.6 %). Pentrating liver injury was recorded in 23 (19.0 %), with equal distribution between the groups: 15(19.0 %) suffered stab wounds, while 8(6.6 %) injured by firearms (Fig. 1). A total of 108(89.2 %) had liver trauma associated with injury >2 body regions, there was no difference between survivors and non-survivors (Table 1). Total of 82(67.8 %) patients had a ISS>34 (Table 2).

**Table 1:** Comparison between clinical characteristics in survivors and non-survivors

Variable	Survivors	Non-survivors	Total	p
	(*n* = 81)	(*n* = 40)	(*n* = 121)	
Male sex^a^	61(75.3 %)	29(72.5 %)	90(74.4 %)	>0.05
Age^b^	35.78 ± 18.54	43.34 ± 12.26	41.36 ± 17.80	>0.05
Penetrating liver injury^a^	16(19.7 %)	7(17.5 %)	23 (19.0 %)	>0.05
Blunt hepatic injury^a^	65 (80.2 %)	33(82.5 %)	98(80.9 %)	>0.05
Associated injury>2 body regions^a^	70(86.4 %)	38(95.0 %)	108(89.2 %)	>0.05

^a^Data are expressed as number of patients and percentages (n, %), ^b^Data are presented as Mean ± Standard Deviation

**Table 2:** Comparison between clinical characteristics in survivors and non-survivors at arrival time and within first 24 h

Variable	Survivors	Non-survivors	Total	p
	(*n* = 81)	(*n* = 40)	(*n* = 121)	
Liver AAST grade III^a^	37(45.7 %)	5(12.5 %)	42(34.7 %)	0.001
Liver AAST grade IV^a^	37(45.7 %)	16(40.0 %)	53(43.8 %)	>0.05
Liver AAST grade V^a^	7(8.6 %)	19(47.5 %)	26(21.5 %)	0.0001
ISS˃34 (arrival)^a^	49(60.5 %)	33(82.5 %)	82(67.8 %)	0.0001
Systolic blood pressure ≤90 mmHg (arrival)^a^	28(34.6 %)	34(85.0 %)	62(51.2 %)	0.0001
GCS˂9 (arrival)^a^	4(4.9 %)	25(62.5 %)	29(23.9 %)	0.0001
AST(U/L) within first 24 h^b^	454.09 ± 130.3	1405.22 ± 605.10	820.32 ± 315.12	0.010
ALT(U/L) within first 24 h^b^	505.13 ± 270.626	905.79 ± 412.385	675.32 ± 189.34	0.033
RBC transfusion (ml) within first 24 h^b^	1500.31 ± 607.46	5810.63 ± 2817.31	2510.03 ± 817.21	0.001

^a^Data are expressed as number of patients and percentages (n, %), ^b^Data are presented as Mean ± Standard Deviation, *AAST* American Association for Surgery of Trauma, *GCS* Glasgow Coma Scale, *ISS* Injury Severity Score, *RBC* Red blood cell

In this study there was 42(34.7 %) patients with liver trauma AAST III, 53(43.8 %) AAST IV and 26(21.5 %) patients with liver trauma AAST V including 4(3.3 %) retrohepatic vena cava and 10(8.3 %) major hepatic veins injury (Table 2). There was a significant difference between survivors 49(60.5 %) with ISS>34 and non-survivors 33(82.5 %) with ISS>34 (*p* = 0.0001) (Table 2). In comparison with the survivors, non-survivors have significantly higher liver AAST grade of injury: there was a statistical significance for the AAST III and AAST V (*p* = 0.001; *p* = 0.0001), while the AAST IV showed the same distribution (*p* > 0.05) (Table 2). Non-survivors showed significant hypotension on arrival (*p* = 0.0001) and lower GCS (*p* = 0.0001) (Table 2). Non-survivors needed significalntly more RBC units transfusions (*p* = 0.001) (Table 2). Non-survivors had significantly higher serum AST and ALT level within first 24 h (*p* = 0.010; *p* = 0.033) (Table 2).

**Fig. 2:**
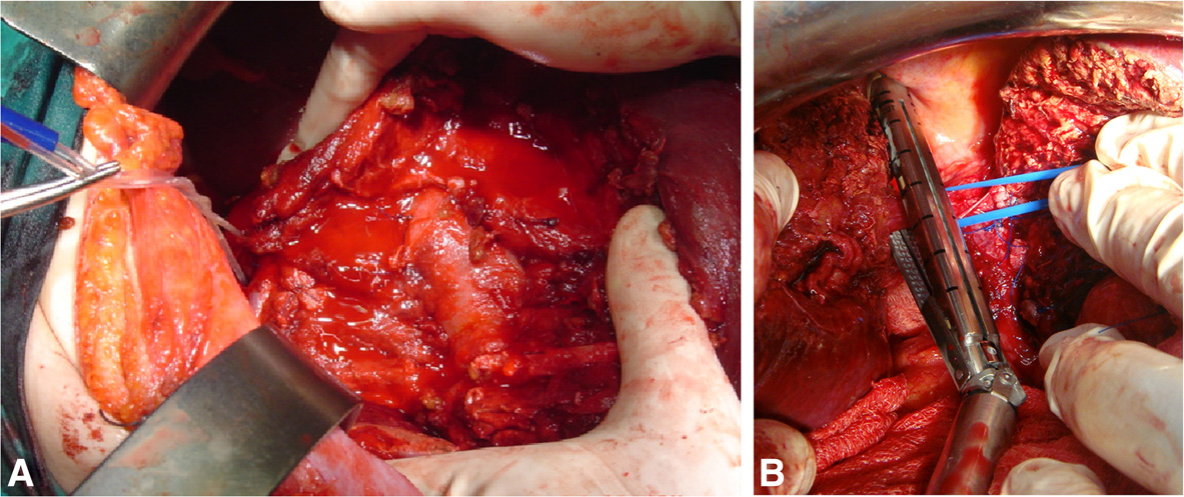
Intraoperative findings in blunt liver trauma. Road traffic accident was the cause of trauma in 40 year old driver with AAST grade V blunt liver injury (**a**). Right hepatectomy: transection of right Glissonean pedicle using endo-GIA vascular stapling device (**b**)

**Fig. 3:**
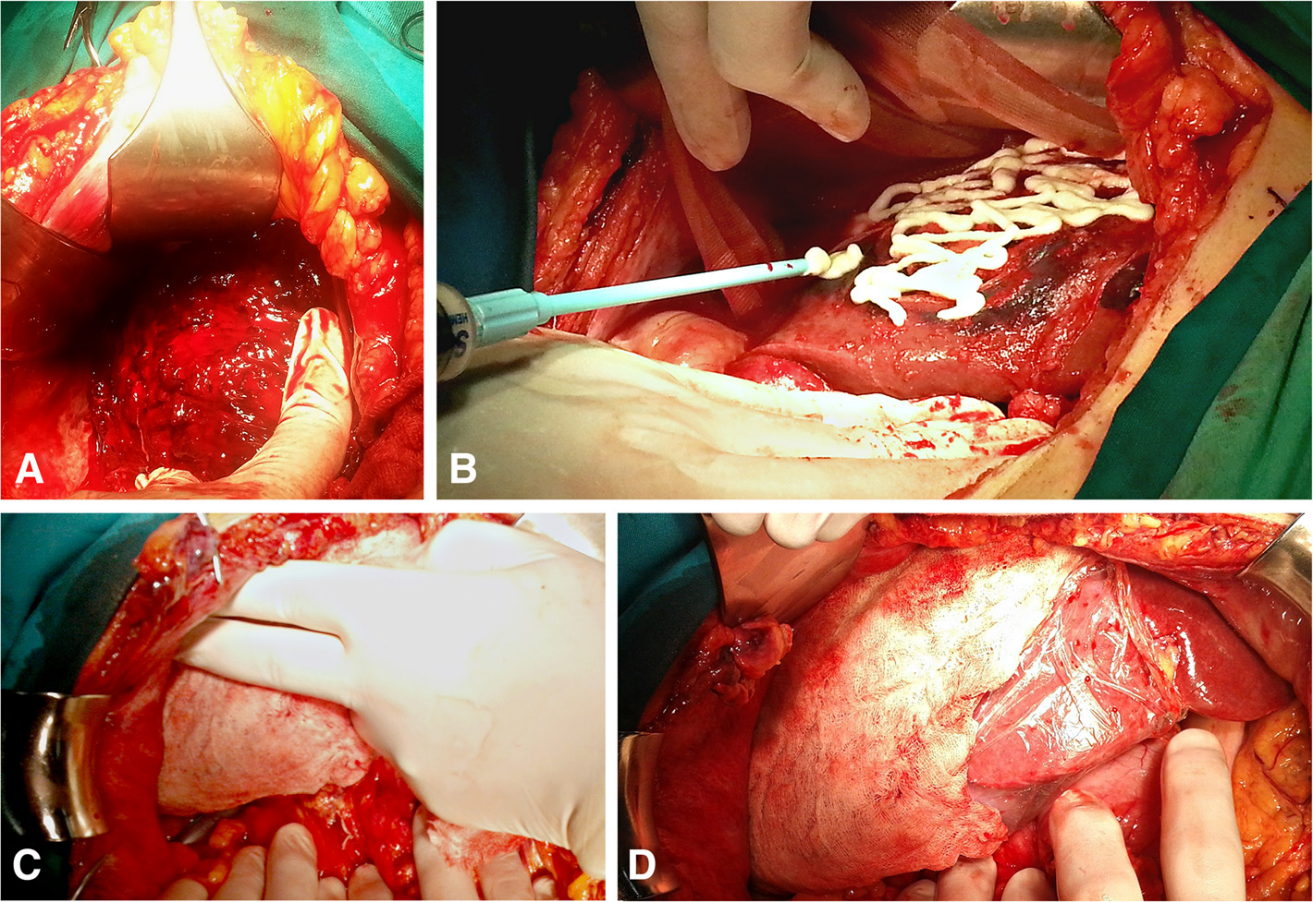
Damage Control Surgery in blunt liver trauma (DCS I –Initial laparotomy). A 41 year old exsanguinating man with AAST grade V blunt liver injury (**a**). In order to control life-threatening hemorrhage emergency laparotomy was followed by direct liver vessel repair with bleeding vessels sutured prior to liver packing and hemostatic fibrin gel on liver surface (**b**). We performed liver packing with four abdominal swabs to provide liver compression (**c**, **d**)

There was no statistically significant difference in the application of surgical approach (*p*= > 0.05) (Table 3). Range of blood removed from peritoneal cavity was 500–1500 ml. Definitive hepatic repair was performed in 62(51.2 %) patients (Table 3). Liver resection was performed in 12(9.9 %) patients: non-anatomic resection in 6(4.9 %) patients and major resection (≥3 Couinauds segments) in 6(4.9 %) (Fig. 1, 2). DCS with perihepatic packing and planned re-laparotomy after 48 h was used in 59(48.8 %) (Table 3). In DC strategy we used different additional procedures in combination with liver packing (Fig. 3).

**Table 3:** Surgical procedures for hepatic hemorrhage control in complex liver trauma

Variable	Survivors	Non-survivors	Total	p
	(*n* = 81)	(*n* = 40)	(*n* = 121)	
Damage control surgery^a^	39(48.1 %)	20(50.0 %)	59(48.8 %)	>0.05
DCS-perihepatic packing + direct parenchyma suture^a^	21(25.9 %)	10(25.0 %)	31(25.6 %)	
DCS-perihepatic packing + liver resection^a^	4(4.9 %)	4(10.0 %)	8(6.6 %)	
DCS-perihepatic packing + RHA ligation^a^	1(1.2 %)	1(2.5 %)	2(1.6 %)	
DCS-perihepatic packing + direct vessel repair^a^	13(16.0 %)	5(12.5 %)	18(14.8 %)	
Definitive hepatic repair^a^	42(51.8 %)	20(50.0 %)	62(51.2 %)	>0.05
Direct parenchyma suture + hemostatic fibrin gel^a^	15(18.5 %)	5(12.5 %)	20(16.5 %)	
Hepatotomy + direct vessel repair, vascular ligation and debridement^a^	23(28.4 %)	5(12.5 %)	28(23.1 %)	
Non-anatomic liver resection^a^	2(2.5 %)	4(10.0 %)	6(4.9 %)	
Major liver resections^a^	2(2.5 %)	4(10.0 %)	6(4.9 %)	
Selective RHA ligation^a^	0(0.0 %)	2(5.0 %)	2(1.6 %)	

^a^Data are expressed as number of patients and percentages (n, %), *DCS* Damage control surgery, *RHA* Right hepatic artery

Most common non-related liver complications were: right-sided exudative pleural effusion in 24(19.8 %) patients, ARDS in 20(16.5 %) and pneumonia in 12(9.9 %) (Table 4). As liver-related complications we recorded: prolonged hemorrhage in 18(14.9 %), bile leak 21(17.3 %), biloma 12(9.9 %), liver abscess 2(1.6 %) and liver failure 1(0.8 %) (Table 4). Regarding complications non-survivors had a significantly prolonged bleeding and higher rate of ARDS (*p* = 0.0001, for all) (Table 4). Survivors had a significantly higher biloma (*p* = 0.014) and pleural effusion (*p* = 0.0001) (Table 4). Eleven (9.0 %) of all patients required re-operation during hospitalization: 9(7.4 %) due to prolonged bleeding and 2(1.6 %) due to uncontrolled bile fistula (Table 4). Other complications were treated with non-surgical approach, in one case the liver failure, patients had associated severe brain and lung injury .

**Table 4:** Postoperative complications in survivors and non-survivors

Variable	Survivors	Non-survivors	Total	p
	(*n* = 81)	(*n* = 40)	(*n* = 121)	
Re-operations^a^	7(8.6 %)	4(10.0 %)	11(9.0 %)	>0.05
Prolonged bleeding^a^	5(6.2 %).	13(32.5 %)	18(14.9 %)	0.0001
Bile fistula^a^	16(19.7 %)	5(12.5 %)	21(17.3 %)	>0.05
Bilom^a^	10(12.3 %)	2(5.0 %)	12(9.9 %)	0.014
Liver abscess^a^	2(2.5 %)	0(0.0 %)	2(1.6 %)	>0.05
Liver failure^a^	0(0.0 %)	1(2.5 %)	1(0.8 %)	>0.05
ARDS^a^	9(11.1 %)	11(27.5 %)	20(16.5 %)	0.0001
Pneumonia^a^	8(9.9 %)	4(10.0 %)	12(9.9 %)	>0.05
Pleural effusion^a^	24(29.6 %)	0(0.0 %)	24(19.8 %)	0.0001

^a^Data are expressed as number of patients and percentages (n,%), *ARDS* Acute respiratory distress syndrome, *MODS* Multiple organ dysfunction syndrome

Mortality was 33.1 %. We recorded statistical significance in terms of ICU and hospital stay (*p* = 0.001; *p* = 0.001) (Table 5). The early trauma-related mortality within the first 24 h after admission was noted in 35 % (Table 5). The cause of mortality in “early period” was massive prolonged bleeding. Among non-survivals 62.5 % died within the first 7 days (Table 5). Patients died in the further course of hospitalization due to late respiratory complications: ARDS and pneumonia.

**Table 5:** Postoperative ICU stay, hospital stay and survival time

Variable	Survivors	Non-survivors	p
	(*n* = 81)	(*n* = 40)	
ICU stay^a^ (days)	2 (1–6)	8.5 (2–18)	0.001
Hospital stay^a^ (days)	30 (15–90)	10 (4–30)	0.001
Died within the first 24 h	/	14(35 %)	
Died within the first 7 days	/	25(62.5 %)	

^a^ICU and hospital stay are presented by median range

## Discusson

In hemodynamically unstable patients with hemorrhage from major liver injury and massive hemoperitoneum on abdominal imaging, the strategy and techniques for bleeding control can be extremely demanding and complex. We presented the results of surgical treatment of 121 trauma patients with severe bleeding liver injuries.

Since Pringle’s publication of inflow vascular control on liver, the primary focus of trauma surgeons was to find the best way to achieve hemo-stasis, bile-stasis and infection control in hepatic injuries [15–23]. Today the focus of trauma surgeons is selection of appropriate patients for operative management, who are the candidates for surgery and when to operate [7, 8]. The general contraindications for none-operative management of liver trauma included the hemodynamic instability, extravasations of intravenous contrast on abdominal imaging, expanding hematoma and grade IV and V liver injury [6, 7]. According to the study conducted by Coimbra et al. nonoperative management has been accepted as treatment of choice only for stable patients with low grade of injury [23]. Fang et al. study of 214 patients with a hepatic injury showed that the independent predictors for the surgical treatment even in hemodynamically stable patients included intraperitoneal contrast extravasation and hemoperitoneum in six compartments on CT scan [22]. FAST is able to sensitively detect hemoperitoneum presented as free fluid in the abdomen and pelvis, but its numerous limitations have been recognized [8, 24]. MSCT is the imaging modality of choice in evaluating hemodynamically stable patients with suspected hepatic injury [7, 22]. Abdominal CT accurately defines the morphology and extent of the hepatic trauma, identifies associated visceral injuries and depicts the amount of hemoperitoneum [7, 25]. It is important to know that life- threatening liver injuries can be detected by MSCT with high sensitivity.

In this study indications for emergency laparotomy were: hemorrhage shock on admission, refractory hemodynamic instability, signs of haemoperitoneum on ultrasound, MSCT findings of the severe liver trauma with contrast extravasation which indicate active hemorrhage. It is important to know that life-threatening liver injuries can be detected by MSCT with high sensitivity. The operative management of liver injuries requires the use of some of the most complex surgical techniques, including extensive hepatotomy with selective deep vessel ligation, hepatorrhaphy, selective hepatic artery ligation, non-anatomic resection and debridement and hepatectomy [6, 7]. The surgical treatments of severe liver injuries in this study were definitive procedure (hepatorrhaphy, hepatotomy with vascular ligation, debridement, selective hepatic artery ligation, liver resection) and DCS with liver packing. The incidence of trauma patients requiring liver packing varies from 5 to 62 % in the literature [6, 21, 26]. We performed perihepatic packing in 48.8 % patients. It is important to recognize that liver packing will not control arterial bleeding and that any bleeding artery should be suture/ligated prior to liver packing. A precise perihepatic packing technique starting with a Pringle manoeuvre and complete liver mobilization, with systematized placement of packs. Strategies for haemorrhage control are more efficient when DCS is associated with appropriate additional strategies such as an effective fluid resuscitation/transfusion protocols, a carefully selected angioembolization and an accurate ICU critical care [13, 19, 27]. According to the Asensio results, improvements in outcome can be achieved using damage control strategy to control hepatic bleeding in patients with severe liver injuries and compromised physiological stage [6]. However, interventional radiology with embolization may play an important role in cases of liver packing followed by re-bleeding. Angiography and angioembolization were not used in our study group which is the limitation of this study. Although this surgical technique was not a predictor of outcome in our study, the question is whether the use of DCS in combination with angioembolization for emergency control bleeding, would contribute to the lower rate of complications and deaths in this study. The reported survival rate associated with packing increased up to 65.5 % as reported by Peitzman et al [2]. Outcome could be improved by combining of venous bleeding control by liver packing and arterial haemorrhage control with angioembolization [2, 5, 6, 28].

Over half of patients surviving grade III–V liver injuries will be at risk for the development of complications [29]. Liver-related complications occur in approximately 20–45 % of patients and include hemorrhage, hemobilia, arteriovenous fistula, pseudo-aneurysm, biloma, bile leak and abscess formation [5, 10, 27, 29]. MSCT and ultrasaund was used in diagnosing specific post- traumatic postoperative complications such as hepatic or perihepatic abscesses or bilomas. Postoperative prolonged hemorrhage can be associated with coagulopathy [5, 10, 27, 29]. While blood transfusion is necessary because of massive bleeding in trauma, it carries many complications [29, 30].

Complex hepatic injuries have a high mortality rate (8,36,38). Our study shows that non-survivors had higher AAST grade of injury, higher AST and ALT level, significant hypotension, higher ISS score and lower GCS on arrival; and significalntly more RBC units transfusions within the first 24 h. Early liver injury-related death is typically secondary to uncontrolled bleeding (20–60 %), which is worsened with attendant coagulopathy, whereas late mortality is usually secondary to multiorgan failure (MOF), Acute Respiratory Distress Syndrom (ARDS) in 27(32.1 %), and Multiple Organ Dysfunction Syndrome (MODS) [6, 29–31]. Previous studies have shown that increased risk of ARDS and MODS has been associated with massive transfusion, which can itself contribute to coagulopathy [30]. In blunt liver trauma mortality also appears to be higher in older patients, those with higher grade injuries, and those with hemodynamic instability on presentation [29–31]. In a series of 144 patients with grade III–V hepatic injuries, uncontrollable bleeding from the liver injury and associated severe splenic injury favors early laparotomy and damage control strategy [31]. Asensio and colleagues showed that predictors of mortality in grade IV–V injuries are related to severe bleeding and include blood loss, number of red cell units transfused, hypothermia, acidosis, and dyasrhythmia [6].

## Conclusion

Prolonged bleeding and amount of blood transfusions are statistically significant predictors of mortality in severe hepatic trauma. All efforts in the treatment of trauma patients with complex liver injuries AAST grade III–V should be directed to the rapid and effective control of liver hemorrhage and taking care of all associated life-threatening injuries.

## Abbreviations

DCS: Damage control surgery
OIS: Organ injury scale
AAST: American association for surgery of trauma
FAST: Focused assessment by ultrasound for trauma
ICU: Intensive care unit
MSCT: Multislice computed tomography
DPL: Diagnostic peritoneal lavage
AST: Aspartate transaminase
ALT: Alanine aminotransferase
ARDS: Acute respiratory distress syndrome
